# Discovery of non-reference processed pseudogenes in the Swedish population

**DOI:** 10.3389/fgene.2023.1176626

**Published:** 2023-05-30

**Authors:** Esmee Ten Berk de Boer, Kristine Bilgrav Saether, Jesper Eisfeldt

**Affiliations:** ^1^Department of Molecular Medicine and Surgery, Center for Molecular Medicine, Karolinska Institutet, Stockholm, Sweden; ^2^Science for Life Laboratory, Karolinska Institutet Science Park, Solna, Sweden; ^3^Department of Clinical Genetics, Karolinska University Hospital, Stockholm, Sweden

**Keywords:** processed pseudogene, whole genome sequencing, population genomic, variant calling, clinical diagnostics

## Abstract

The vast majority of the human genome is non-coding. There is a diversity of non-coding features, some of which have functional importance. Although the non-coding regions constitute the majority of the genome, they remain understudied, and for a long time, these regions have been referred to as junk DNA. Pseudogenes are one of these features. A pseudogene is a non-functional copy of a protein-coding gene. Pseudogenes may arise through a variety of genetic mechanisms. Processed pseudogenes are formed through reverse transcription of mRNA by LINE elements, after which the cDNA is integrated into the genome. Processed pseudogenes are known to be variable across populations; however, the variability and distribution remains unknown. Herein, we apply a custom-designed processed pseudogene pipeline on the whole genome sequencing data of 3,500 individuals; 2,500 individuals from the thousand genomes dataset, as well as 1,000 Swedish individuals. Through these analyses, we discover over 3,000 pseudogenes missing from the GRCh38 reference. Utilising our pipeline, we position 74% of the detected processed pseudogenes—allowing for analyses of formation. Notably, we find that common structural variant callers, such as Delly, classify the processed pseudogenes as deletion events, which are later predicted to be truncating variants. By compiling lists of non-reference processed pseudogenes and their frequencies, we find a great variability of pseudogenes; indicating that non-reference processed pseudogenes may be useful for DNA testing and as population-specific markers. In summary, our findings highlight a great diversity of processed pseudogenes, that processed pseudogenes are actively formed in the human genome; and that our pipeline may be used to reduce false positive structural variation caused by the misalignment and subsequent misclassification of non-reference processed pseudogenes.

## 1 Introduction

The majority of the human genome is non-coding. There is a wide array of non-coding genomic features, many are essential for the proper functioning of the genome (Gloss and Dinger, 2018). Although non-coding DNA constitutes the majority of the genome, these regions remain understudied. Pseudogenes are one type of non-coding feature. Pseudogenes are typically inactive features, carrying a close sequence similarity to functional genes (Zhang et al., 2003). Novel pseudogenes are pseudogenes that are not present in the reference genome. These are abundant and have widespread effects on genotyping and other sequencing based diagnostic methods (Mandelker et al., 2016; Zhang et al., 2017).

The human genome contains 8,000 pseudogenes, but there is a lot of variation between different species (Vanin, 1985; Zhang et al., 2003). There are two major classifications of pseudogenes: duplicated (non-processed) and processed pseudogenes. Duplicated pseudogenes arise via gene duplications or unequal crossing over followed by mutations that render the copied gene non-functional. Often non-processed pseudogenes maintain an intact intron-exon structure. Processed pseudogenes, or retrotransposed genes, arise from the reverse transcription of mRNA into cDNA mediated by long interspersed nuclear element (LINE) retrotransposons (Esnault et al., 2000). This results in the integration of a cDNA copy of the gene into the genome at a different location than the parent gene (Vanin, 1985; Esnault et al., 2000; Zhang et al., 2003). Processed pseudogenes thus lack introns, promoter and enhancer sequences. Instead, they often have a poly-A-tail. Furthermore, processed pseudogenes often have L1 transposition markers, such as target-site duplications (TSDs) (Zhang et al., 2017). Despite the lack of promoters and other mediators of expression, processed pseudogenes can contribute to the generation of new genes and the evolution of the genome (Kaessmann, 2010; Ciomborowska et al., 2013). Classification of pseudogenes is of importance in a diagnostic context, as pseudogenes may affect the accuracy of variant detection in their parent gene (Zhang et al., 2017). Furthermore, in whole genome sequencing (WGS), the reads originating from pseudogenes absent from the reference genome (i.e., novel pseudogenes) will map to the parent gene. This results in reads with too large insert sizes, which are interpreted as variation by structural variant detection tools (Mandelker et al., 2016). As such, novel pseudogenes can affect the clinical analysis of patients, which reduces the speed and accuracy of diagnosis. Many pseudogenes have a disease-relevant gene as their parent gene. For this reason, accurately annotating novel pseudogenes is an important step in the genomic testing of patients. In this paper, we present a pipeline that can find processed pseudogenes missing from a reference genome, referred to as novel pseudogenes, in a diagnostic context. By applying the pipeline to WGS data from the 1,000 genomes project (1KGP) (The 1000 Genomes Project Consortium et al., 2015) and the SweGen cohort (Ameur et al., 2017), we find 3,021 processed pseudogenes that are missing from GRCh38 (Schneider et al., 2017). Previous work has attempted to characterise the presence of processed pseudogenes in the 1,000 genomes project using low coverage (∼5X) WGS data and/or WES (Ewing et al., 2013; Schrider et al., 2013; Zhang et al., 2017). These studies characterised various novel pseudogenes highlighting the importance of this topic.

Using our pipeline, we find that novel pseudogenes are present in nearly 2/3rd of individuals. Some novel pseudogenes are present in all tested populations, whereas others are population specific. We confirm the accuracy of pseudogene calls by extracting coverage patterns of the parent genes of common novel pseudogenes. These patterns confirm the presence of novel pseudogenes.

## 2 Materials and methods

### 2.1 Processen pipeline

The Processen pipeline (https://github.com/J35P312/Processen) finds novel pseudogenes by extracting mapped read pairs, such that the reads are mapped within the same gene but on different exons, with a larger than expected insert size. The reads are extracted using samtools. These reads are then aligned to the GenCode transcriptome using the Salmon sequence aligner (Patro et al., 2017). Salmon is run using the following command:

salmon quant -i <transcript_index> -l A -1 <R1> -2 <R2> -o <output_directory> --validateMapping

Where the transcript index is an index of the reference transcripts, R1 and R2 are the read pairs previously extracted by the pipeline and output directory is the directory of the output. Next the pipeline will search the output files for transcripts present in the output. If Salmon reports more than 20 reads in a transcript, and the coverage across the transcript is at least 3X, that transcript is considered a processed-pseudogene candidate (Supplementary Figure S1). The 3X threshold was chosen to reflect that many SV callers require at least three reads or fragments to report SV (Rausch et al., 2012; Chen et al., 2016; Eisfeldt et al., 2017); The 20 reads threshold is to ensure that the pipeline finds exons chains and not deletions overlapping exons or something similar.

### 2.2 Delly

To obtain variant calls for each of the genomes in 1KGP and the SweGen consortium Delly (v. 0.8.7) was used (Rausch et al., 2012). The command was ran as:

delly call -o < bcf > -g <GRCh38> < bam>

Where bcf is the output file containing the variants found by Delly. GRCh38 is the reference genome, and the bam file is the bam file of one individual in the 1KGP or SweGen consortium.

For four common pseudogenes the number of deletion calls by Delly was counted, the high priority deletion calls were counted separately.

### 2.3 VEP

To annotate variant calls for each of the genomes in 1KGP and the SweGen consortium VEP (v. 107.0) was used (McLaren et al., 2016). The input for the command was bcf files generated by Delly. The command was ran as:

vep --cache --dir $VEP_CACHE --offline -i <bcf> -o <vcf> --vcf --assembly GRCh38 --per_gene --format vcf --no_stats --force_overwrite

Where VEP_CACHE is the path to the VEP cache, bcf are the files generated by Delly as described above, vcf is the output vcf. For more information see the manual (https://www.ensembl.org/info/docs/tools/vep/script/vep_options.html#basic).

### 2.4 Positioning of processed pseudogenes

To obtain the position of an identified novel pseudogene, we search for SVs in the annotated vcf file obtained as previously described. To find SV calls representing the insertion of processed pseudogenes, we first extract SV calls such that one position maps close to the start or end of the parent gene, and the other position must map to another chromosome or at least the parent gene length away from the first position. To obtain the unique insert sites these locations were filtered based on distance. If the distance between two insert sites is larger than 500 bp the insert sites were annotated as unique. If two insert sites were within 500 bp of one another, they were collapsed into a single insert site. See the Processen pipeline (https://github.com/J35P312/Processen) for more information.

### 2.5 Visualization of processed pseudogenes

The circos plot showing the insertion sites of novel pseudogenes were created using circos (v. 0.69.9), available via (http://circos.ca/software/).

### 2.6 Whole genome sequencing data

Two genomic datasets were used for analysis: the WGS dataset from the 1KGP consortium and the SweGen consort (Ameur et al., 2017).

The WGS data from 1KGP was obtained from the UPPMAX computational infrastructure. Further information about the 1KGP dataset can be obtained from 1KGP and UPPMAX websites (The 1000 Genomes Project Consortium et al., 2015).

The SweGen cohort contains WGS data of 1,000 Swedish individuals. All samples were obtained from blood. Each genome has an average 30X coverage. Sequencing was done using a polymerase chain reaction-free 120-bp paired-end library. Further information about the SweGen cohort can be found (Ameur et al., 2017).

### 2.7 Reference genome pseudogene data

Pseudogenes present in the reference genome were extracted from the comprehensive gene annotation of GRCh38 from GenCode (https://www.gencodegenes.org/human/).

### 2.8 Gene ontology analysis

Novel pseudogenes from the 1KGP and SweGen datasets were pooled and analysed using PantherGO (http://pantherdb.org/). GO enrichment was tested using standard settings, and GO term count was downloaded for further processing. This analysis was repeated in the same way for reference pseudogenes.

### 2.9 Sequence feature enrichment analysis

The genomic position of several genome features were obtained from the gencode primary assembly (v38) and indexed according to Tabix instructions (Li, 2011). The intersection of these genomic features and pseudogene insert sites were found using Tabix. The statistical significance of the enrichment of the various genomic features was found via a Monte Carlo stimulation followed by a binomial test: for 10,000 iterations, 1 chromosome was randomly chosen based on its length, then 1 random insert site was generated for each random chromosome the intersection between genomic features and the randomly generated insert sites were obtained using Tabix. Following this, the fraction of insertions on a specific genomic feature was calculated, and a binomial test was used to test if the observed fraction significantly differs from the randomly generated one. This test was repeated separately for 1KGP novel pseudogenes and SweGen novel pseudogenes. Significance was corrected using a Bonferroni correction.

### 2.10 Extracting coverage of parent genes

Coverage of the parent genes of novel pseudogenes was obtained using Samtools depth (v.1.16) (Li et al., 2009), for the region of the gene of interest as obtained from the gencode primary assembly (v38). The command was run as:

Samtools depth -a -o <output_file> -r <gene_region> <cram_file>

The depths per position were normalised to the average depth of chromosome 1 for each individual and then multiplied by two to obtain a good estimate of the diploid genome coverage. Then the depth per position was averaged over all individuals who share the same pseudogene or those who do not have the pseudogene of interest as a control.

Each intron and exon’s start and stop sites were obtained from the gencode primary assembly (v38). The average depth per intron and exon was computed using the average normalised depth per position and then plotted.

This analysis was repeated for *SKA3* and *PRKRA*.

## 3 Results

### 3.1 Novel pseudogenes in 3,500 genomes

We applied the Processen pipeline to analyse 2,500 genomes from 1KGP and 1,000 genomes from the SweGen database. In total we find 2,215 novel pseudogenes with 55 unique parent genes in 1KGP (Supplementary Table S1) and 806 novel pseudogenes with 13 unique parent genes in SweGen (Supplementary Table S2). The non-redundant set of unique parent genes between both groups is 59.

Novel pseudogenes originating from *PRKRA* and *SKA3* were present in almost all populations. Most other parent genes only had novel pseudogenes in one or very few populations. In this way, the novel pseudogenes result in considerable variability between populations. Among the 55 parent genes, ∼70% are exclusive to a single population. In this way, the retroduplication of genes can result in large genomic differences between different population.

**FIGURE 1:**
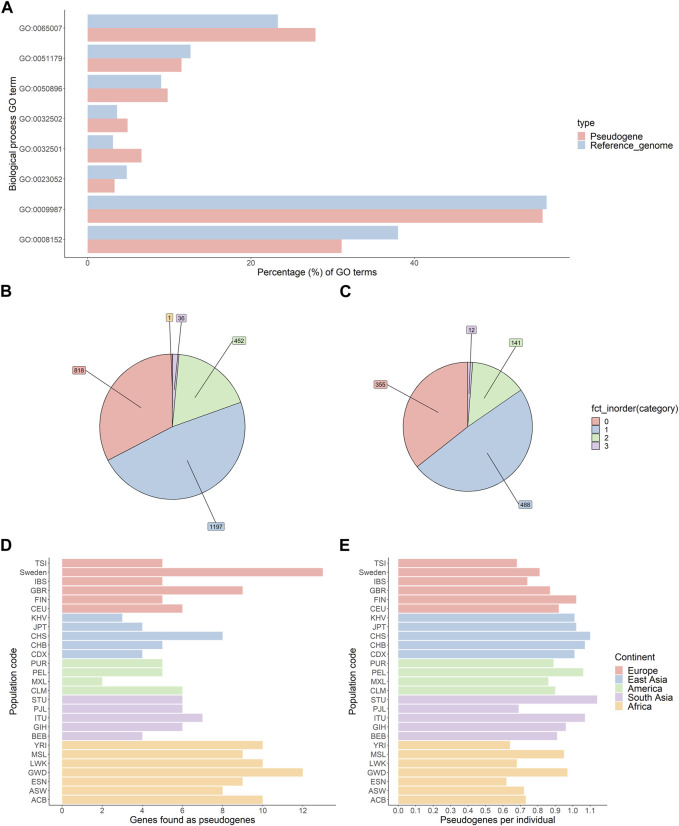
Characterisation of novel pseudogenes identified by the Processen pipeline. **(A)** Comparison of the distribution of biological function gene ontology (GO) terms for reference and novel pseudogenes. **(B,C)** Number of novel pseudogenes per individual in the 1KGP genomes **(B)** and SweGen genomes **(C)**. **(D)** Number of unique parent genes per population. **(E)** Average number of pseudogenes per individual per population.

As only expressed genes can generate processed pseudogenes, the function and expression profile of parent genes can give clues about the timing of pseudogene generation. The parent genes of all novel pseudogenes were pooled and checked for gene ontology (GO) term enrichment using PantherGO. No GO terms were significantly enriched. We compared this result to parent genes of pseudogenes present in the GRCh38 reference genome. In this analysis also, no GO terms were significantly enriched. Both novel pseudogenes and reference pseudogenes have a similar distribution of GO terms, as found by PantherGO (Figure 1A).

For both 1KGP and the SweGen consortium, 2/3rd of individuals carry at least one novel pseudogene (Figures 1B, C).

Next, we compared the number of novel parent genes per population. The average number of novel pseudogenes per genome is slightly less than 1.0 for each population. Notably, African populations appear to have a greater diversity of pseudogenes than other populations (Chi square test; *p*-value AFR vs. EUR = 0.014, *p*-value AFR vs. SAS = 0.58, *p*-value AFR vs. EAS = 0.027) (Figure 1D, E).

A larger number of unique parent genes are found in Swedish genomes than in other genomes. This difference may be due to the larger sample size of Swedish genomes than other populations. The increase in the number of novel pseudogenes shows that testing more genomes results in finding more rare novel pseudogenes, indicating a large diversity of rare pseudogenes within populations.

### 3.2 Processed pseudogenes preferentially insert into specific genome features

**FIGURE 2:**
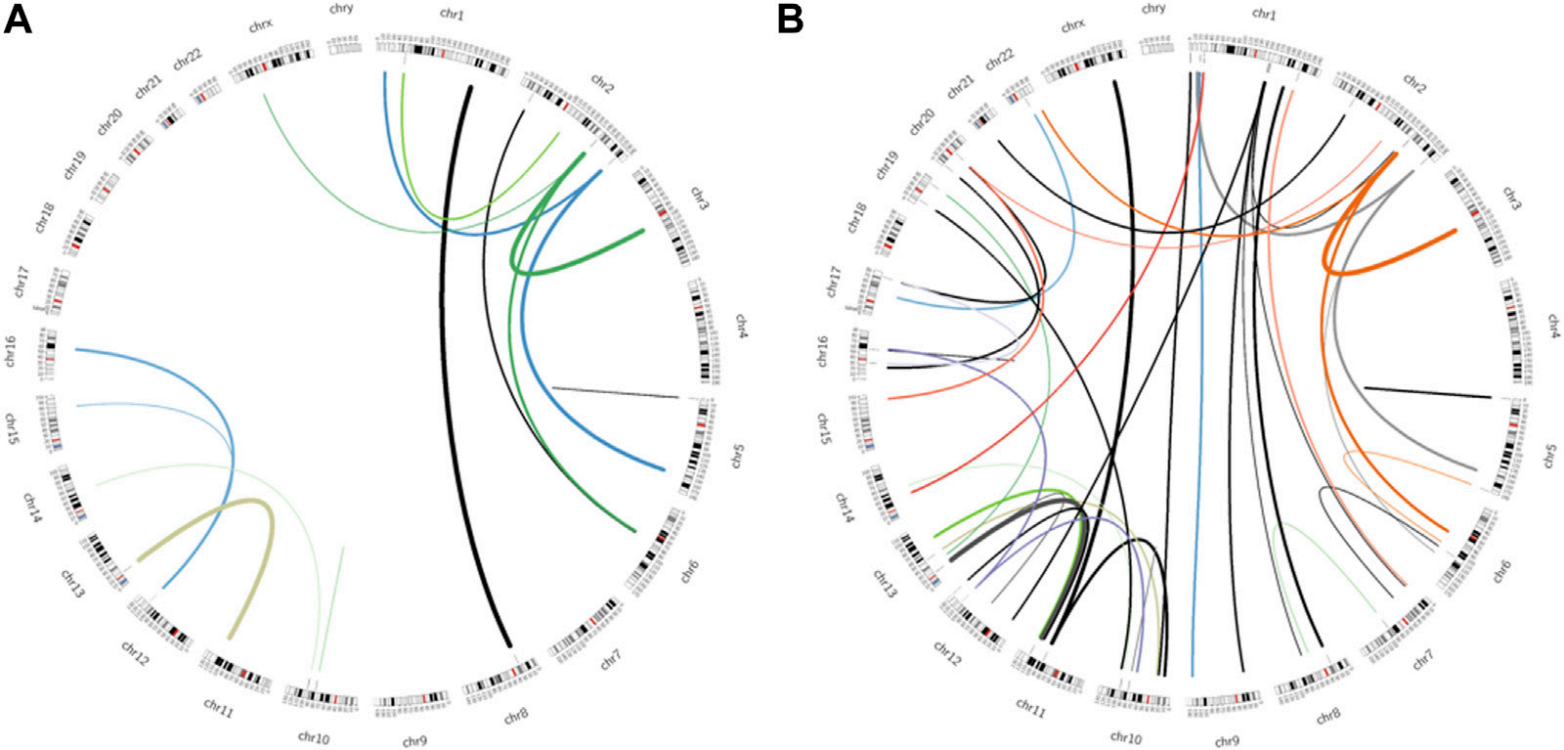
Circos plot showing the path between parent gene location and the insert locations of novel pseudogenes. **(A)** SweGen insert sites. **(B)** 1KGP insert sites.

Following identification, the positioning of the novel pseudogenes was analysed using the Processen pipeline. Approximately 74% of novel pseudogenes could be positioned in the genome. We found 55 unique genomic positions for the 1KGP novel pseudogenes and 20 for the SweGen pseudogenes. Several positions are shared between 1KGP and SweGen novel pseudogenes (Figure 2). Common pseudogenes may be inserted at several genomic positions, indicating that there have been multiple events where the parent gene was retro transposed to form a new processed pseudogene.

The novel pseudogene are inserted evenly across the genome (Supplementary Figure S2). Notably, novel pseudogenes appear to be enriched within segmental duplication and reference processed pseudogenes (Supplementary Figure S2).

### 3.3 Processed pseudogenes show a characteristic coverage pattern

**FIGURE 3:**
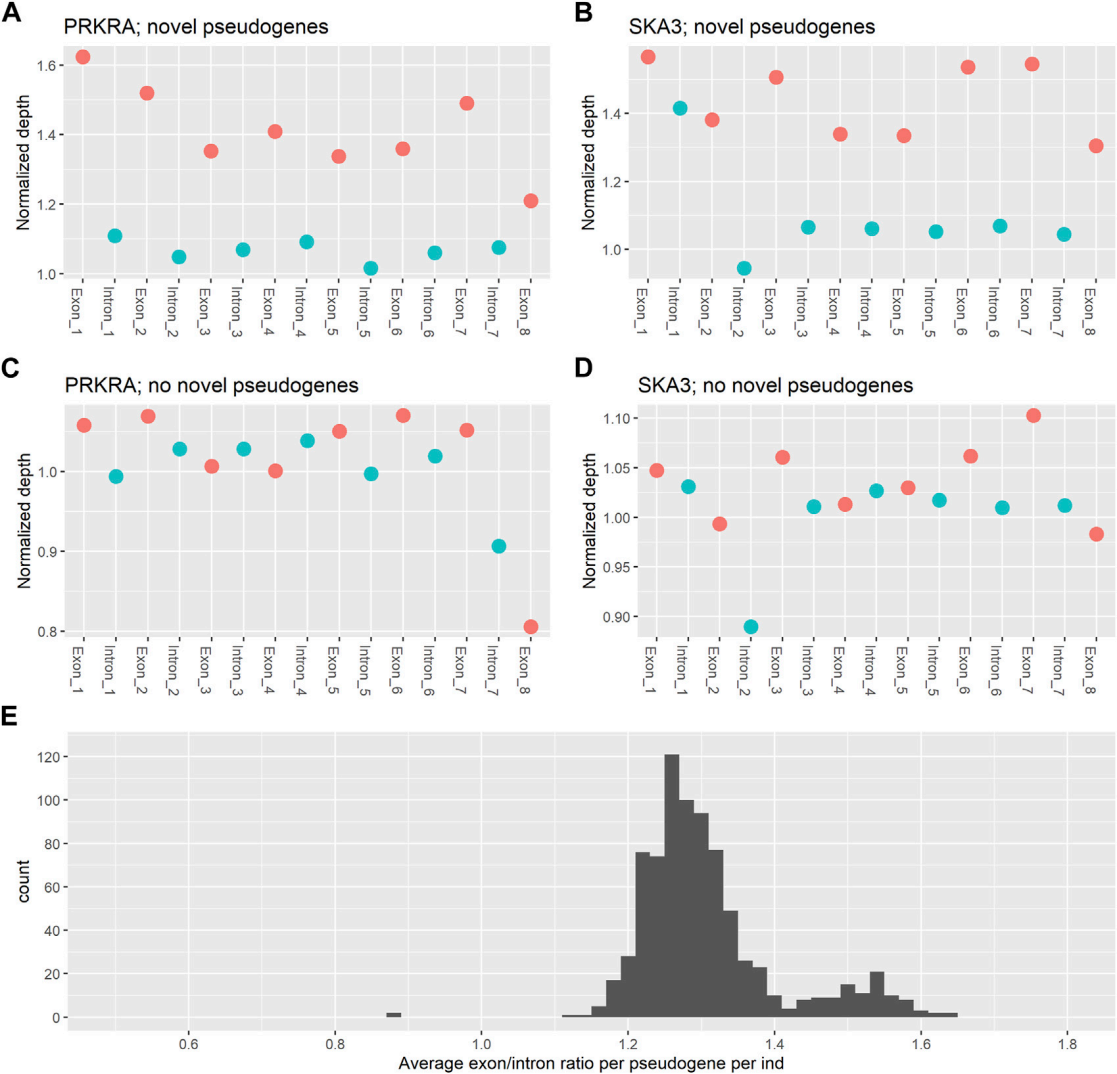
Average normalised coverage of parent genes. **(A)** The average coverage per intron and exon for the PRKRA gene for individuals that have a novel pseudogene originating from PRKRA. **(B)** The average coverage per intron and exon for the SKA3 gene for individuals that have a novel pseudogene originating from SKA3. **(C)** The average coverage per intron and exon for the PRKRA gene for individuals that do not have a novel pseudogene originating from PRKRA. **(D)** The average coverage per intron and exon for the SKA3 gene for individuals that do not have a novel pseudogene originating from SKA3. **(E)** A histogram of the average exon/intron ratio per pseudogene per individual.

To verify the presence of novel processed pseudogenes, the coverage patterns of parent genes with a predicted novel pseudogene were examined for the two most common novel pseudogenes within the SweGen consortium. For *PRKRA* (ENST00000325748.9) (Figure 3A; Supplementary Figure S3) and *SKA3* (ENST00000298260.8) (Figure 3B; Supplementary Figure S3), the average exon coverage is higher than the average intron coverage. This is as expected if a novel pseudogene originating from these genes is present. We compared these coverage patterns to the coverage patterns of the *PRKRA* and *SKA3* genes in individuals that do not have novel pseudogenes originating from these genes (Figures 3C, D; Supplementary Figure S3). In individuals with no novel pseudogene from *PRKRA* or *SKA3* the coverage of introns and exons is approximately equal. This confirms that the novel pseudogenes from the Processen pipeline are truly novel pseudogenes and not other sequencing artefacts.

The coverage ratio between exons and introns in the novel pseudogenes found by the pipeline presented in this paper is again confirmed when plotting the distribution of average exon/intron ratios per individual per parent gene (Figure 3E). The majority of ratios are centred around 1.3, showing that exons in the predicted novel pseudogenes have ∼30% higher coverage than the introns. There is also a smaller peak with coverage ratios around 1.5. This peak can be explained by individuals with 2 novel pseudogene copies of the same parent gene.

Overall, a coverage that is ∼30% higher shows that there are three exon copies for every two intron copies. This coverage pattern is characteristic of a parent gene having a processed pseudogene, as the pseudogene will introduce an extra copy of each exon into the genome, but no introns will be present. In this way, these results confirm that the pipeline successfully identifies novel processed pseudogenes.

### 3.4 Novel pseudogenes occur in disease-related genes and are mistaken for deletions by structural variant callers

Clinical variant caller Delly often mistakes novel pseudogenes for deletions. When running Delly using the BAM files of individuals with novel pseudogenes as input, novel pseudogenes are indicated as intronic and occasionally exonic deletions. The average number of deletion calls for each of the most common novel pseudogenes is indicated in Table 1. All pseudogenes have a large average number of deletion calls, and all can result in high-priority deletion calls.

**TABLE 1:** The average number of deletion calls by Delly for four common novel pseudogenes.

	Average deletion calls	Average high-priority deletion calls	Max. deletion calls	Max. high priority deletion calls
*SKA3*	14	1	20	5
*PRKRA*	15	0	21	1
*FBXL5*	22	0	27	1
*MFF*	12	0	16	1

As some of these novel pseudogenes are disease-related genes (e.g., *SKA3*), each of these erroneous deletion calls increased the time till diagnosis for genomic diagnostics as they have to be manually checked. This shows the importance of accurately classifying novel pseudogenes in patient genomes.

## 4 Discussion

We present Processen, an efficient pipeline for the detection of novel processed pseudogenes in clinical settings. Using the Processen pipeline on the 2,500 genomes from 1KGP and the 1,000 genomes from the SweGen consortium we identify 3,021 novel processed pseudogenes, originating from 59 unique parent genes, that are missing from the GRCh38 reference genome. These novel pseudogenes are located throughout the entire genome. The GRCh38 reference genome lacks several common, as well as many rare, population specific processed pseudogenes.

Previous work has attempted to characterise the presence of processed pseudogenes in the 1,000 genomes project using low coverage (∼5X) WGS data and/or WES (Ewing et al., 2013; Schrider et al., 2013; Zhang et al., 2017). Notably, we report fewer novel pseudogenes than a previous study by Zhang et al. (2017), who report 15,642 retroduplications originating from 503 unique parent genes based on the 1KGP data of 2,533 individuals. This study used the GRCh37 reference genome, which may contain fewer pseudogenes than GRCh38. Moreover, this study employs a different method of pseudogene identification, using whole exome sequencing (WES) data rather than WGS, the authors map unmappable exome sequences to exon-exon junction libraries to generate the retroduplication calls (Zhang et al., 2017). Technical reasons can thus explain the difference in results, and the true amount of novel pseudogenes can only be accurately determined with long-read sequencing.

It is unclear why some parent genes, such as *PRKRA* and *SKA3*, are more prevalent among the novel pseudogenes than other genes. Either, these genes may be more prone to generate processed pseudogenes, such patterns could be explained through the expression levels of the gene, or the presence of sequence similarity with LINE elements, which would make these sequences more likely to be targeted by LINE retrotransposons (Esnault et al., 2000). Alternatively, these pseudogenes may be older, and therefore widely spread through different populations and individuals. However, we note that these, common novel pseudogenes have several insert sites, indicating that there have been several occasions in which the parent gene was retro transposed to form a new pseudogene. We therefore suggest that some genes may be more likely to generate processed pseudogenes, though it remains unclear why. Overall, the lack of significant GO terms shows that there may be a broad timeframe of pseudogene generation during development, or the developmental stage during which most pseudogenes are generated has a broad expression profile.

The novel pseudogenes vary widely between populations, possibly resulting in phenotypic differences between the populations (Stranger et al., 2007; Kidd et al., 2008; Conrad, 2013). The large diversity of novel pseudogenes within and between populations indicate that processed-pseudogenes could be used in genetic testing and as population markers. However, for this purpose, it would be essential to characterise many novel pseudogenes accurately.

The presence of rare novel pseudogenes indicates that new processed-pseudogenes are formed continuously. This underwrites the importance of individual approaches to genome sequencing and variant calling rather than relying on a single reference genome. Moreover, we see that the reference genome lacks pseudogenes from all populations, which indicates that being able to find novel pseudogenes in patient genomes is essential for patients from any population.

We show that novel pseudogenes preferentially insert into non-coding regions of the genome. This is as expected, as large inserts into active genes and regulatory regions may result in negative selection. However, to confirm this result, more insert sites should be identified. It has been suggested previously that long read sequencing can aid in the identification and positioning of non-reference sequences (Eisfeldt et al., 2020). We again suggest here that the accuracy of novel pseudogene identification and localisation can be increased by using long read sequencing.

Overall, there is an abundance of novel pseudogenes within human genomes, and each of these novel pseudogenes can result in delayed diagnosis during genetic testing. This highlights the importance of being able to find and classify novel pseudogenes prior to variant calling.

A more diverse reference genome is required (Eisfeldt et al., 2020), however, we find that novel pseudogenes differ significantly between populations and individuals. The majority of novel pseudogenes are rare, and each sequenced individual is likely to add new parent genes. As such, merely making the reference genome more diverse will not solve the problem of non-reference pseudogenes in genomic testing. These difficulties may be overcome by using graph reference data structures (Paten et al., 2017), though even then it will be difficult to incorporate all diversity originating from the novel pseudogenes that will be discovered with increased genomic testing.

In summary, we present a pipeline that can accurately identify novel pseudogenes in genomes within a diagnostic timeframe. We tested this pipeline on 2,500 genomes from 1KGP and 1,000 genomes from the SweGen consortium. We find 3,021 novel pseudogenes originating from 59 parent genes. We confirm the pipeline accurately finds novel pseudogenes by plotting the coverage profiles of parent genes of common novel pseudogenes. All in all, we conclude that greater diversity in the reference genome is necessary to capture the genetic diversity between individuals. However, even more importantly we conclude that individual approaches to genomic testing and variant calling are important within a diagnostic context.

## Data Availability

The original contributions presented in the study are included in the article/Supplementary Material, further inquiries can be directed to the corresponding author.
