# Supplementary material for: Discovery of non-reference processed pseudogenes in the Swedish population
Source: Front Genet. 2023 May 30;14:1176626. doi: 10.3389/fgene.2023.1176626 (PMC10267823; doi:10.3389/fgene.2023.1176626)
Supplement: Supplementary file 1 [file DataSheet1.docx]

Supplementary Material

Discovery of non-reference processed pseudogenes in the Swedish population

Esmee ten Berk de Boer^*^, Kristine Seather Bilgrav, Jesper Eisfeldt

*** Correspondence:** Corresponding Author: esmee.ten.berk.de.boer@ki.se

# Supplementary tables

## Supplementary table 1

**Supplementary table 1: Novel pseudogenes identified in the 1KGP database by the Processen pipeline**. The table shows the parent gene name, the number of genomes in which the novel pseudogene was found and the populations in which the novel pseudogene was found

| **Parent gene name** | **Number of individuals with novel pseudogene** | **Population with novel pseudogene** |
| --- | --- | --- |
| *PRKRA* | 1155 | GBR, FIN, CHS, PUR, CDX, CLM, IBS, PEL, KHV, ACB, GWD, PJL, ESN, BEB, MSL, STU, ITU, CEU, YRI, CHB, JPT, LWK, MXL, ASW, TSI, GIH |
| *SKA3* | 676 | GBR, FIN, CHS, PUR, CDX, CLM, IBS, PJL, KHV, ACB, PEL, GWD, BEB, MSL, ESN, STU, ITU, CEU, YRI, CHB, JPT, LWK, MXL, ASW, TSI, GIH |
| *FBXL5* | 99 | CLM, ACB, GWD, ESN, MSL, YRI, LWK, ASW |
| *MFF* | 70 | GBR, FIN, PUR, ACB, PEL, GWD, PJL, ESN, MSL, ITU, YRI, YRI, JPT, LWK, ASW, GIH |
| *TMEM126B* | 69 | PUR, ACB, CDX, GWD, MSL, ESN, YRI, LWK, ASW |
| *USP28* | 22 | ACB, GWD, ESN, MSL, YRI, LWK |
| *SARAF* | 16 | GBR, FIN, PUR, PEL, PJL, BEB, ITU, CEU, GIH |
| *MTMR2* | 13 | FIN, GBR, CLM, IBS, CEU, TSI |
| *FOXK2* | 10 | CHS, CHB, JPT |
| *SPTLC1* | 9 | ESN, MSL, YRI, ASW |
| *POLR2C* | 7 | ACB, MSL, ESN, LWK, ASW |
| *RXYLT1* | 5 | YRI, LWK, ASW |
| *C20orf27* | 4 | CLM, ITU, TSI |
| *C14orf93* | 4 | PJL, ITU |
| *AHCY* | 4 | STU |
| *PSRC1* | 4 | STU |
| *SECISBP2* | 3 | GWD, MSL |
| *RCBTB1* | 3 | GWD |
| *ANKLE2* | 3 | GIH |
| *ZDHHC11* | 2 | GBR, TSI |
| *PPP1CC* | 2 | GBR, CEU |
| *INO80C* | 2 | CDX |
| *MT1E* | 1 | GBR |
| *SH3RF1* | 1 | GBR |
| *DDX21* | 1 | CHS |
| *JTB* | 1 | CHS |
| *SFTPA1* | 1 | CHS |
| *SPATA7* | 1 | CHS |
| *SSU72* | 1 | CHS |
| *PPP2R5A* | 1 | CLM |
| *MAZ* | 1 | IBS |
| *KIF17* | 1 | IBS |
| *ECSIT* | 1 | PEL |
| *DAP3* | 1 | ACB |
| *ANKIB1* | 1 | ACB |
| *MICAL1* | 1 | ACB |
| *STX12* | 1 | GWD |
| *POLK* | 1 | KHV |
| *PRKRAG1* | 1 | PJL |
| *MRPS7* | 1 | GWD |
| *TALDO1* | 1 | GWD |
| *PACSIN3* | 1 | GWD |
| *RACK1* | 1 | STU |
| *NAA25* | 1 | ESN |
| *MRPL9* | 1 | BEB |
| *TEAD2* | 1 | STU |
| *PDLIM1* | 1 | ITU |
| *NDUFS1* | 1 | CHB |
| *EMID1* | 1 | CEU |
| *TSR1* | 1 | CHB |
| *FEZ2* | 1 | YRI |
| *TIRAP* | 1 | LWK |
| *RSPH1* | 1 | LWK |
| *AP2M1* | 1 | GIH |
| *PPP1R21* | 1 | YRI |

## Supplementary table 2

**Supplementary table 2: Novel pseudogenes identified in the SweGen database by the processen pipeline.** The table shows the parent gene name, the number of genomes that the novel pseudogene was identified in, and whether or not the pseudogene was also found in the 1KGP database.

| **Parent gene name** | **Number of individuals with novel pseudogene** | **Present in 1KGP genomes (n)** |
| --- | --- | --- |
| *SKA3* | 264 | Yes (676) |
| *PRKRA* | 470 | Yes (1155) |
| *PPP1CC* | 2 | Yes (2) |
| *SARAF* | 31 | Yes (16) |
| *MFF* | 9 | Yes (70) |
| *MTMR2* | 20 | Yes (13) |
| *CZIN* | 1 | No |
| *HLA-DRB1* | 3 | No |
| *WDR43* | 2 | No |
| *ZDHHC11* | 1 | Yes (2) |
| *SFTPA1* | 1 | Yes (1) |
| *SKA1* | 1 | No |
| *PDLIM* | 1 | Yes (1) |

# Supplementary figures

## Supplementary figure 1

**
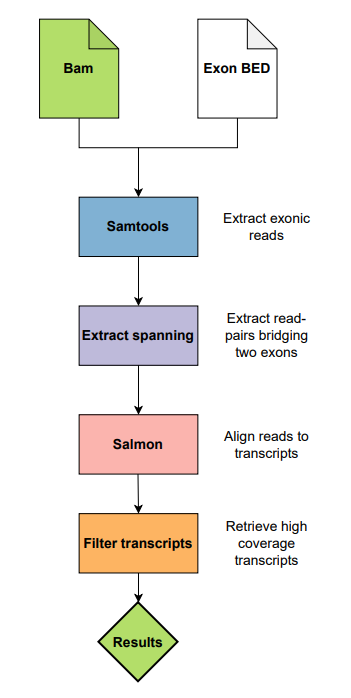
**

Schematic of the workflow of the Processen pipeline to find novel pseudogenes.

## Supplementary figure 2


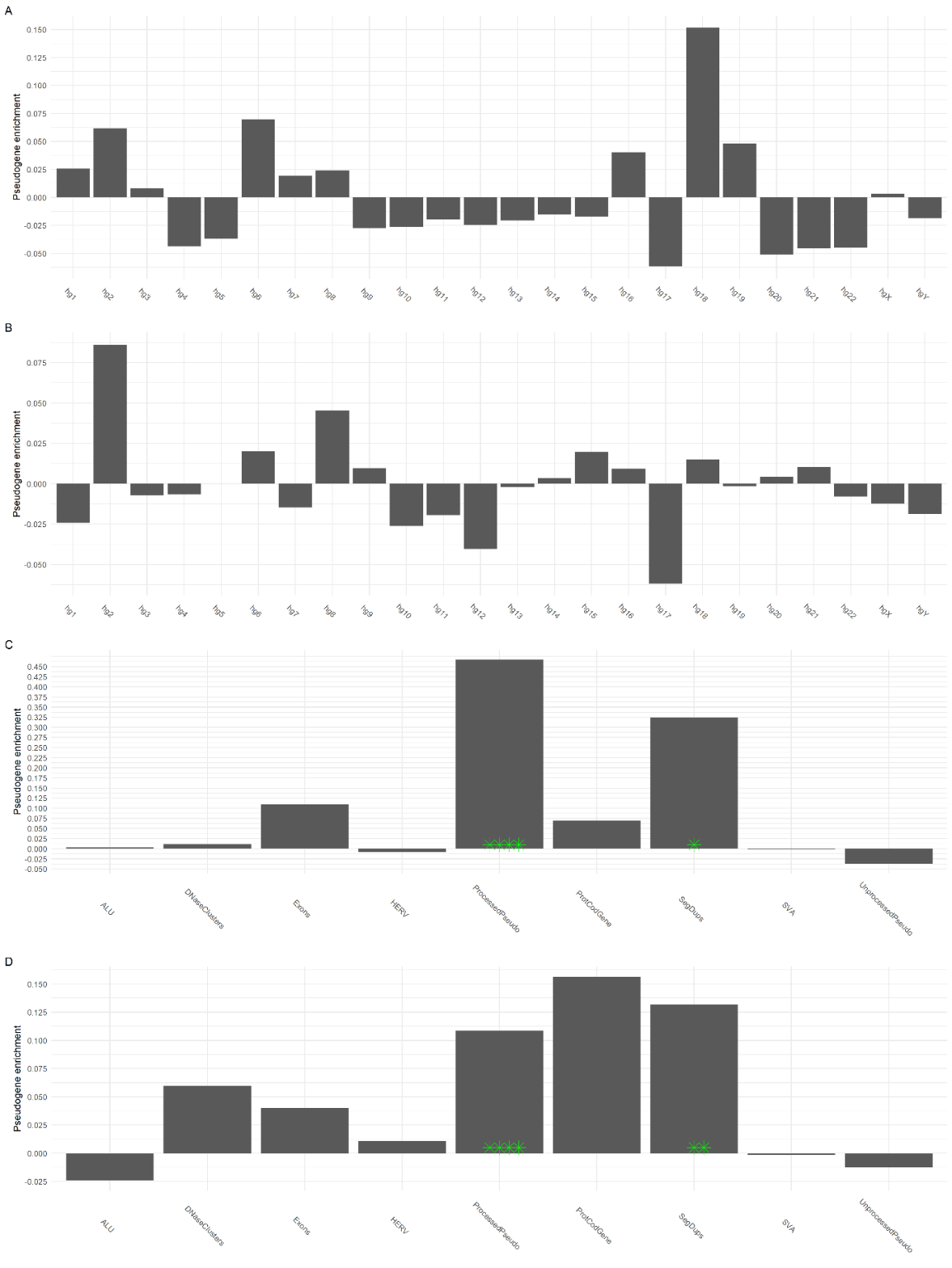


Genomic feature enrichment of pseudogene insert sites and chromosomal enrichment of pseudogene insert sites. A,B) Chromosomal enrichment of A) 1KGP insert sites, B) SweGen insert sites. C,D) Genome feature enrichment of C) 1KGP pseudogene insert sites, D) SweGen pseudogene insert sites. Significance (with Bonferoni correction): *: p<0.05, **: p<0.01, ***: p< 0.001, ****: p<0.0001.

## Supplementary figure 3


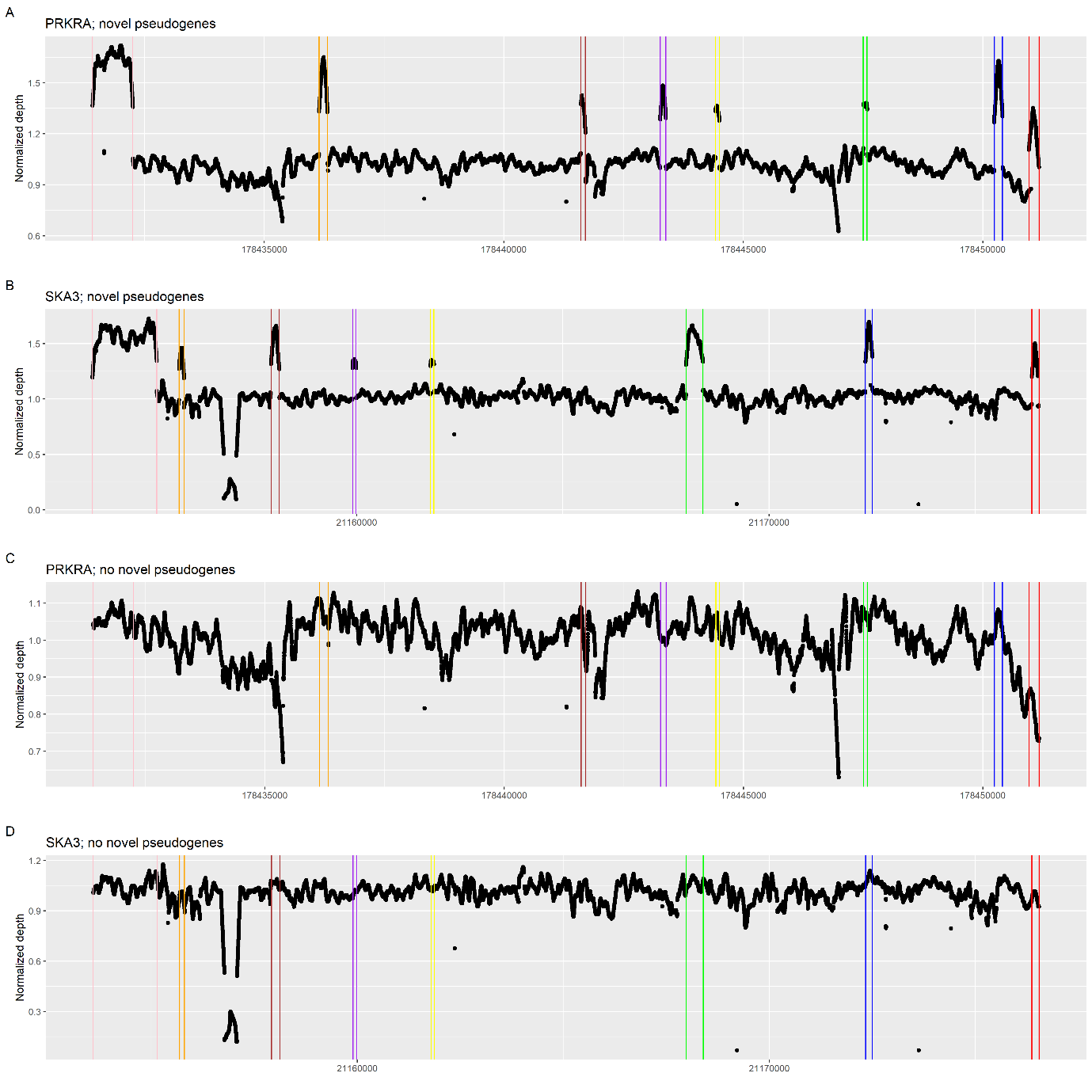


Average normalised coverage of parent genes. Exon boundaries are indicated by coloured bars. Bars of the same colour represent the start site and stop site of a single exon. A) The average coverage per nucleotide for the PRKRA gene for individuals that have a novel pseudogene originating from PRKRA. B) The average coverage per nucleotide for the SKA3 gene for individuals that have a novel pseudogene originating from SKA3. C) The average coverage per nucleotide for the PRKRA gene for individuals that do not have a novel pseudogene originating from PRKRA. D) The average coverage per nucleotide for the SKA3 gene for individuals that do not have a novel pseudogene originating from SKA3.
